# Maximising the impact of qualitative research in feasibility studies for randomised controlled trials: guidance for researchers

**DOI:** 10.1186/1745-6215-16-S2-O88

**Published:** 2015-11-16

**Authors:** Alicia O'Cathain, Pat Hoddinott, Simon Lewin, Kate Thomas, Bridget Young, Joy Adamson, Yvonne Jansen, Nicola Mills, Graham Moore, Jenny Donovan

**Affiliations:** 1 ScHARR, Sheffield, UK; 2 University of Stirling, Stirling, UK; 3 Norwegian Knowledge Centre for the Health Services, Oslo, Norway; 4 University of Liverpool, Liverpool, UK; 5 University of York, York, UK; 6 Behavioural and Societal Sciences, Delft, the Netherlands; 7 University of Bristol, Bristol, UK; 8 Cardiff University, Cardiff, UK

## 

Feasibility studies are increasingly undertaken in preparation for randomised controlled trials in order to explore uncertainties and enable trialists to optimise the intervention or the conduct of the trial. Qualitative research can be used to examine and address key uncertainties prior to a full trial. We present guidance that researchers, research funders and reviewers may wish to consider when assessing or undertaking qualitative research within feasibility studies for randomised controlled trials. The guidance was compiled by an expert panel of 10 researchers from three countries who have experience of this endeavour. The guidance consists of 16 items within five domains: research questions, data collection, analysis, team work and reporting. Example items are: Consider the range of qualitative methods and approaches that might be used to address the key feasibility questions, including dynamic or iterative ones which allow learning from early qualitative research to be implemented before undertaking further qualitative research within the feasibility study; Consider the timing of analysis, which might be in stages in a dynamic approach. This guidance may help researchers to consider the full range of contributions that qualitative research can make in relation to their particular trial. The guidance may also help researchers and others to reflect on the utility of such qualitative research in practice, so that trial teams can decide when and how best to use these approaches in future studies.

